# Long-Term Cognitive and Functional Outcomes Following Postoperative Delirium and Liberal Fluid Fasting in Elderly Trauma Patients: A Prospective Single-Centre Study

**DOI:** 10.3390/jcm15135316

**Published:** 2026-07-07

**Authors:** Patricia Knabe, Janine Allmendinger, Tobias Haas, Max Knabe, Lina Lenninger, Anne-Marie Just, Boris Holzapfel, Carl Neuerburg, Roland Tomasi, Thomas Saller

**Affiliations:** 1 Department of Anaesthesiology, LMU Munich University Hospital, 81377 Munich, Germany; 2 Department of Anaesthesiology, Intensive Care, Emergency and Pain Medicine, University Hospital Würzburg, 97080 Würzburg, Germany; 3 Max Planck Institute for the Study of Crime, Security and Law, 79100 Freiburg im Breisgau, Germany; 4 Clinical Nursing Research and Quality Management Unit, LMU Munich University Hospital, 81377 Munich, Germany; 5 Department of Orthopaedic and Trauma Surgery, Musculoskeletal University Center Munich (MUM), LMU Munich University Hospital, 81377 Munich, Germany; 6 Department of Orthopaedics and Trauma Surgery, Heinrich-Heine-University, 40225 Düsseldorf, Germany

**Keywords:** cognitive decline, elderly trauma patients, follow-up, functional decline, liberal fluid fasting concept, long-term, quality of life, postoperative delirium

## Abstract

**Background**: Postoperative delirium (POD) is a frequent and serious complication in elderly surgical patients. Liberalising preoperative fluid fasting has been shown to reduce its incidence. However, evidence on long-term cognitive and functional outcomes following POD or liberal fluid fasting remains limited. **Objectives**: This study investigates whether POD and different fluid fasting regimens are associated with changes in cognitive performance and activities of daily living twelve months after surgery. **Methods**: As a follow-up to the prospective ‘LFFgertrud’ trial, 89 geriatric patients were contacted by phone twelve months after elective trauma or orthopaedic surgery (March 2023–February 2024). Participants completed four validated questionnaires assessing cognition and everyday functioning: the Short Blessed Test (SBT), EQ-5D-5L, Barthel index (BI), and the Informant Questionnaire on Cognitive Decline in the Elderly (IQCODE). **Results**: The hypotheses regarding the effects of POD and fluid fasting on cognitive and functional outcomes were not statistically supported. In contrast, neurodegenerative disease was strongly associated with poorer outcomes in three of the four follow-up measures (SBT: β = 1.27, *p* = 0.01; EQ-5D-5L: β = 1.43, *p* < 0.001; BI: β = −1.63, *p* < 0.001), and polymedication also emerged as a relevant predictor. Although not statistically significant, descriptive trends indicated that patients who developed POD showed lower cognitive performance and reduced quality of life at twelve months. **Conclusions**: POD and fluid fasting duration were not significantly associated with long-term cognitive or functional outcomes one year after hospitalisation. The findings highlight the importance of considering pre-existing neurocognitive disease and polypharmacy when assessing the risk of adverse long-term outcomes in older patients. Due to the multifactorial nature of recovery in this population, further research involving larger sample sizes is required in order to gain a better understanding of the factors that influence long-term outcomes.

## 1. Introduction

Postoperative delirium (POD) is a frequent and serious complication in older surgical patients, with reported incidence rates ranging from 4.5% to 41.2% among orthopaedic surgery patients [1]. Delirium is a complex and transient neurological condition that affects consciousness, attention, cognition and motor function. It is a neuropsychiatric complication thought to involve neuroinflammation, neurotransmitter dysregulation and blood–brain barrier dysfunction. These mechanisms may not only contribute to acute cognitive disturbances, but also to longer-term cognitive and functional decline. This provides a rationale for investigating the association between POD and subsequent outcomes [2]. It is common in surgical patients over the age of 65 and is often associated with serious outcomes [3,4]. It is linked to a longer hospital stay, increased mortality, and a risk for long-term cognitive impairment [3,5,6,7,8,9,10]. Previous delirium impairs the ability to cope with everyday life and may lead to a functional decline or even necessitate admission to nursing homes [11]. Furthermore, POD increases staff workload and leads to longer hospital stays and higher healthcare costs [3,6,12].

As there is no therapy for delirium yet, establishing preventive measures in the daily clinical routine plays an important role in fighting this neuropsychiatric pathology. Current guidelines recommend the routine screening of at-risk patients using validated assessment tools such as the confusion assessment method (CAM) or the 4 ‘A’s Test (4AT), available in many validated national versions (e.g., German, Greek) [13]. They also recommend the early recognition of delirium symptoms and the implementation of multicomponent, non-pharmacological prevention strategies. Diagnosis is based on disturbances in attention, awareness, and cognition that are acute and fluctuating in onset. Management primarily focuses on identifying and treating the underlying causes, optimising supportive care and minimising precipitating factors [14].

Previous studies have identified various POD risk factors, including advanced age, comorbidities, polypharmacy, malnutrition, dehydration, and cognitive decline [1,7,15]. Our primary study investigated one modifiable risk factor that was mentioned by Radtke et al. [16]: the role of the duration of preoperative fluid fasting in the development of POD.

Preoperative fasting times frequently exceed the European Society of Anaesthesiology and Intensive Care’s (ESA) recommended 2 h, with patients often fasting substantially longer [17,18,19]. Prolonged fluid fasting is associated with increased postoperative stress and anxiety and may contribute to the development of postoperative delirium [19,20,21]. Exactly this connection was investigated in the baseline trial “LFFgertrud”. The use of liberal fluid fasting has been proposed as a strategy to reduce perioperative dehydration and physiological stress. As postoperative delirium has been linked to subsequent cognitive and functional decline, optimising perioperative care through measures such as liberal fluid fasting could have implications that extend beyond the immediate postoperative period. However, little is known about whether such interventions can influence long-term cognitive and functional outcomes. In a 365-day telephone-administered follow-up, we further explored the association between cognitive and functional changes after delirium or after liberal fluid fasting.

As described, patients who have suffered from POD are more likely to develop cognitive decline in the future, and the risk of institutionalisation rises after delirium [5,7,9,12,22]. Furthermore, there is evidence for a poorer quality of life and functional decline after POD [23]. This longitudinal study provides rare long-term data on liberal fluid fasting, twelve months after its implementation and adds to the limited existing research.

Objectives: This study aims to investigate whether there are differences in cognitive performance and activities of daily living twelve months postoperatively between patients (1) who experienced postoperative delirium and those who did not, and (2) between patients who followed a liberal fluid fasting regimen and those who adhered to restrictive fasting guidelines.

Four different cognitive and functional tests are designed to address this question of interest: the Short Blessed Test (SBT), the EQ-5D-5L, the Barthel index (BI) and the Informant Questionnaire on Cognitive Decline in the Elderly (IQCODE). This leads to the following hypotheses:

**Hypothesis** **1:**  *People who have suffered from POD will differ significantly in their SBT, EQ-5D-5L, BI and IQCODE scores from people who did not suffer from POD.*

**Hypothesis** **2:**  *Participants’ preoperative fasting time of liquids (FTL) is associated with their SBT, EQ-5D-5L, BI and IQCODE score at a 12-month follow-up.*

## 2. Materials and Methods

‘Liberal Fluid Fasting in Geriatric Patients for the Reduction of delirium and Neurocognitive Deficits’ (LFFgertrud) is a prospective before-and-after single-centre trial, performed on four different wards at the LMU University Hospital Munich Campus Großhadern, an academic tertiary hospital in southern Germany. Ethical approval and consultative support for the study were granted by the Ethics Committee of LMU (reference number: 21-0903). The study was prospectively registered in the German Clinical Trials Register (DRKS00026801) by established research transparency standards. A total of 150 patients were enrolled in two cohorts for systematic observation before and after implementing a liberal fluid fasting (LFF) between February 2022 and February 2023. The aim was to investigate the impact of a liberal preoperative fluid fast on the occurrence of postoperative delirium as the primary endpoint. One of the secondary endpoints was care requirements, and a cognitive assessment was administered by telephone after twelve months. This paper focuses on follow-up data as a key cornerstone for investigating the potential long-term effects of post-operative dehydration (POD) and liberal fluid fasting. Key aspects of the baseline study are discussed to provide context.

Inclusion criteria included age ≥ 70 years, elective surgery, duration of anaesthesia ≥ 60 min, and signed informed consent. Exclusion criteria included preoperative delirium, known cognitive impairment, artificial feeding, brain or heart surgery, gastrointestinal disease, palliative care, insufficient knowledge of German, and no informed consent. Although alcohol and substance use were assessed at baseline, only a negligible number of participants reported relevant use. These variables were therefore not included in further analyses. Medication burden was considered through an assessment of polypharmacy.

The data collection itself was carried out by qualified staff at the LMU Campus Großhadern and stored on an internal server.

**Figure 1:**
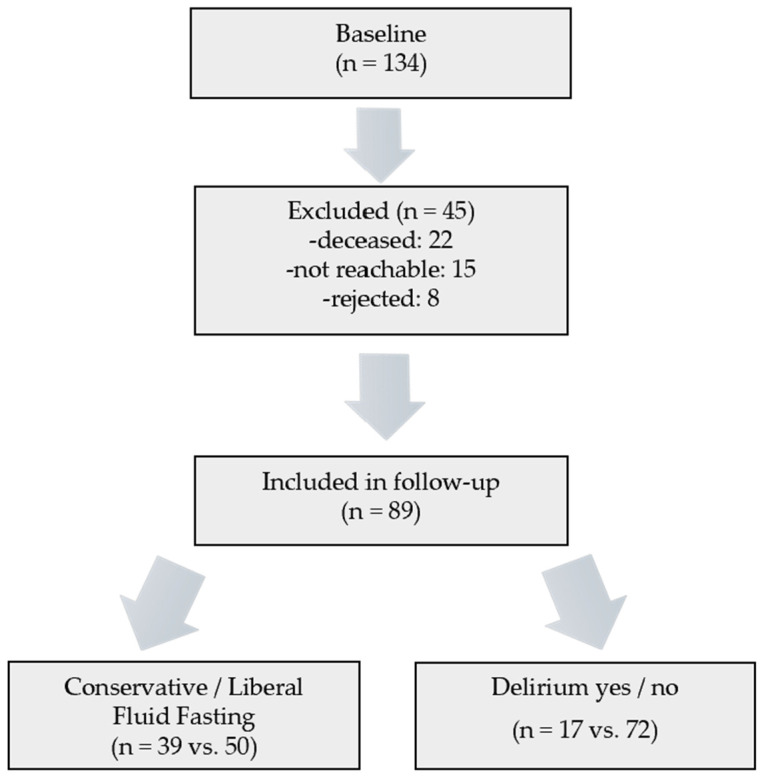
Overview of the participants’ follow-up and failure to follow-up.

Sample: Out of 150 participants, 134 were ultimately analysed; the other 16 were not screened further due to external reasons, such as surgery cancellation. The follow-up sample consists of the same patients as the baseline study. In total, 134 patients were contacted during the period between 1 March 2023 and 28 February 2024. In total, 22 patients passed away, 15 were not reachable, and eight refused to participate, so the initial sample shrank to 89 (Figure 1). If we were unable to reach participants by telephone, we attempted to contact them by letter or through their relatives or family doctor. If all else failed, we tried to find their contact details through the residents’ registration office if they had given consent and agreed to participate in the follow-up. Identity was verified by having the participants provide their last name, first name, and date of birth over the phone.

Of these 89 patients, 39 were in the first cohort, so they did not adhere to any specific fasting rules; of these, 13 suffered from POD. In total, 50 patients were in the second group; they followed a liberal fluid fasting concept, and four suffered from POD.

We performed a sensitivity analysis to determine the effect sizes we could confidently estimate based on the available sample. Based on our sample of *N* = 89, we were able to robustly test the effects of size f-squared of 0.107 at a level of significance alpha α = 0.05 and a desired test power of 1 − β = 0.80. The following results have to be interpreted in the light of that test sensitivity.

Design: We performed a prospective follow-up to the LFFgertrud project 365 days after admission, which was designed as a longitudinal study with a second measurement time. It is worth noting that the data of the study are nested into blocks due to the random assignment of patients to two conditions in the baseline study.

Several tests were performed to measure the impact of POD and liberal fluid fasting on the patients’ outcomes concerning postoperative survival, care requirements and cognitive decline. In this setting, POD and FTL are the predictor variables, and the four tests are the outcome variables. Several other potential predictors were also included from the baseline data and analysed.

Material and Procedure: To facilitate the interpretation of the present follow-up analyses, the baseline assessment of the cohort is summarised below.

Patients who met the inclusion criteria and provided written informed consent were enrolled in the baseline cohort. At this stage, they underwent a structured assessment comprising validated questionnaires on cognitive function, frailty, functional status, quality of life, nutritional status and symptoms of dehydration. Information on comorbidities, medication, laboratory parameters and self-reported fluid and food intake was also collected. Two consecutive fasting protocols were applied. Participants in the first cohort followed the standard fasting regimen of solid food up to six hours and clear fluids up to two hours before anaesthesia. Participants in the second cohort followed a liberal fluid fasting protocol until 06:00 or until transfer to the operating theatre, depending on the scheduled surgery time. During the perioperative period, a sonographic assessment of gastric content was performed and analysed as part of the baseline cohort assessment [24]. Postoperatively, patients were screened daily for five consecutive days for delirium using the 4AT by both the nursing staff and the study team. To improve data quality, 4AT results were cross-checked with clinical progress notes and documented delirium diagnoses in the electronic medical records. In addition, patients were asked about their actual preoperative fasting time for fluids and solids as well as their subjective well-being.

At twelve months, we interviewed the patients who had given informed consent by telephone to assess cognitive function, activities of daily life and functional independence. At the same time, we also inquired about their state of health, living situation and general well-being. Therefore, patients were called during a period of six weeks before or after their date of admission, and one year later. Four different tests were conducted: The Short Blessed Test (SBT), the European Quality of Life 5 Dimensions 5 Level Version (EQ-5D-5L), the Informant Questionnaire on Cognitive Decline in the Elderly (IQCODE) and the Barthel index. For detailed information on the tests, see Table A1 in the Appendix A.

The four instruments were selected to capture complementary aspects of cognitive and functional health. The Short Blessed Test (SBT) assesses current cognitive performance, while the Informant Questionnaire on Cognitive Decline in the Elderly (IQCODE) provides an informant-based evaluation of cognitive decline over time. Together, they offer both cross-sectional and longitudinal perspectives on cognitive status [25,26].

Functional status was assessed using the Barthel index (BI) and the EQ-5D-5L. The BI measures independence in basic activities of daily living, whereas the EQ-5D-5L evaluates broader health-related quality of life across domains such as mobility, self-care, pain/discomfort, and anxiety/depression. Thus, the BI focuses on functional capacity, while the EQ-5D-5L captures the individual’s overall health perception [27,28].

Using these instruments together enabled us to assess participants in multiple dimensions, including cognitive performance, cognitive decline, functional independence, and health-related quality of life. Each instrument provided unique information, minimising redundancy and offering a thorough characterisation of cognitive and functional outcomes. Using the SBT, IQCODE, BI and EQ-5D-5L together was intended to provide complementary information on current cognitive status, cognitive decline, functional independence and health-related quality of life, reflecting the multidimensional nature of cognitive and functional health.

To minimise practice effects, alternate test versions were used at follow-up where available, and the long interval between assessments made relevant learning effects unlikely. The informant-based IQCODE further reduced the risk of recall bias.

Analysis Plan: To test our predictions, we conducted a series of ordinary least squares (OLS) regressions, using POD and FTL as predictors and selected baseline parameters as controls to assess their impact on test performance. To avoid multicollinearity and suppressor effects, follow-up tests were not included as control variables, since they measure similar constructs. We further assessed multicollinearity as well as other model assumptions and report on them below. Each model was tested against the conventional level of significance of α = 0.05. To account for multiple comparisons across the four outcome variables of interest, we adjusted each model using the Benjamini–Hochberg false discovery rate correction [29]. Statistical analysis was performed using IBM SPSS Statistics version 29 (IBM Corp., Armonk, NY, USA) and R version 2023.12.1+402 (R Core Team, Posit Software, Boston, MA, USA, 2024).

## 3. Results

Pre-processing of data and preliminary analysis.

Before starting the analysis, we had to calculate the EQ-5D-5L sum score. The EuroQol Group recommends converting the number combination into an index [30]. Due to licensing restrictions, we only calculated the sum consistent with the baseline study, thus enabling a comparison and analysis of individual items separately. Furthermore, we calculated the index of the IQCODE for a better understanding of the values.

### 3.1. Descriptive Statistics

Demographics were mostly consistent with the baseline as they were based on the same sample (Table 1).

Key baseline parameters relevant for later comparisons were summarised in Table A2 and Table A3 as means and standard deviations. They were divided into ‘delirium no’ or ‘delirium yes’, and into ‘conservative’ or ‘LFF’, as this is more consistent with the hypothesis. The variable ‘liberal fluid fasting’ was being analysed as ‘fasting time liquids’ (FTL).

Now the results of the 12-month follow-up are presented below; see all results in Table 2, Table A4, Table A5 and Table A6.

The analysis begins with the Short Blessed Test (SBT). Patients who developed delirium showed poorer cognitive performance than those without delirium, in both fasting cohorts.

**Figure A1:**
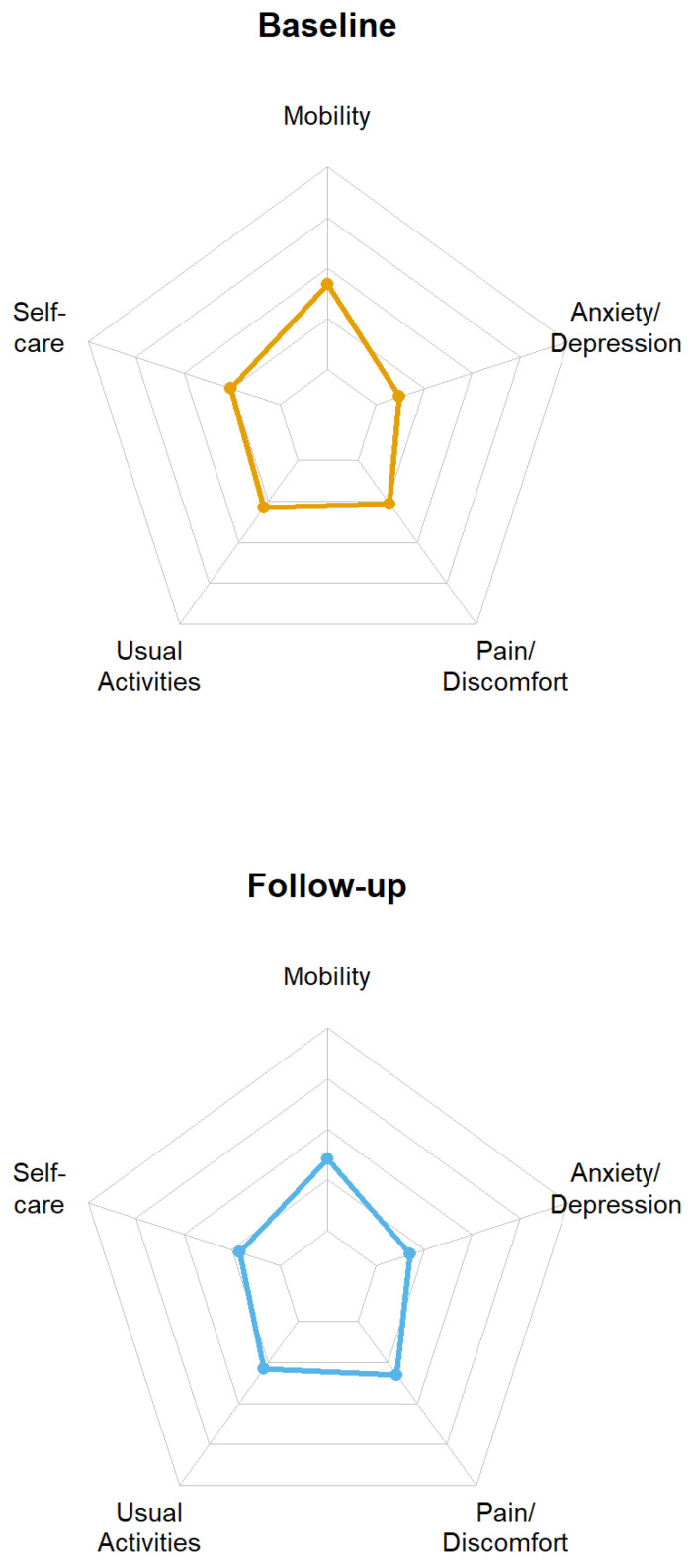
Chart summary of the EQ-5D-5L items for baseline and follow-up.

**Figure A2:**
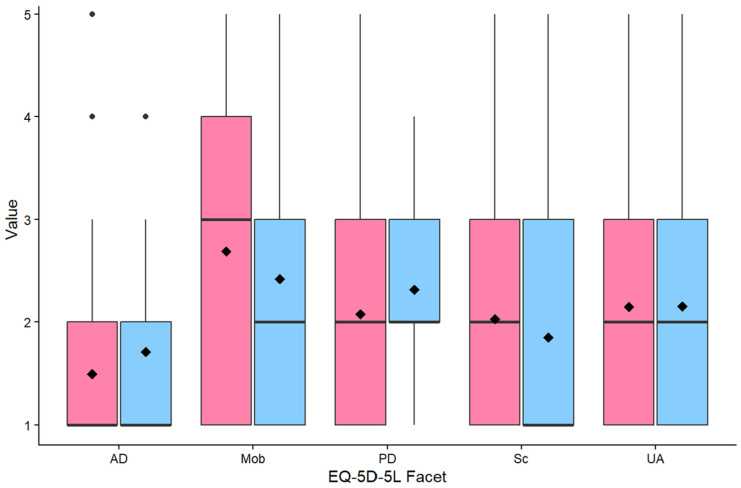
Single EQ-5D-5L facets of the baseline (pink) and the follow-up (blue).

Continuing with the EQ-5D-5L, delirium was associated with reduced performance on this test in all categories compared to patients without delirium. Similar results were observed during their hospital stay twelve months prior (Table A3). The exact decoding of each element and the comparison to the baseline are shown in Figure A1 and Figure A2.

The Barthel index also showed a similar picture to the previous tests, with cognitive performance being weaker in the group of formerly delirious patients.

The last test was the IQCODE, which has been converted into the IQCODE score. The difference between patients with and without delirium was only slight in this case.

However, Table 2 suggests that patients with liberal fluid intake and no delirium tended to perform better in all tests. Overall, patients who experienced postoperative delirium showed poorer cognitive and self-care outcomes at twelve months. Although there are no correlations with FTL, the tests did correlate with each other, as can be seen in Table A6. Potential multivariate associations will be further investigated in the inferential statistics.

### 3.2. Inferential Analysis

In this section, the outcome of the inferential analysis is presented (see Table 3, Table 4, Table 5 and Table 6).

To test the relationship between the Short Blessed Test and POD or FTL, we assessed an OLS regression with POD and FTL as main predictors and SBT as the response. The model assumptions for this model were widely fulfilled, and multicollinearity was below the conventional threshold (VIF < 5) [31]. This model suggested no significant effects of POD or FTL on SBT (POD: β = −0.52 [−1.25, 0.20], *p* = 0.285; FTL: β = −0.00 [−0.22, 0.21], *p* = 0.98).

However, some of the control variables did show an effect. Neurodegenerative disease showed a positive effect on SBT, CCI had a small positive effect on SBT, as well as the EQ-5D-5L. The MoCA had a small negative effect on SBT. These results did not support the main hypotheses but suggest that there might be a correlation between neurocognitively impaired patients and the results of a cognitive test such as the SBT.

Continuing with the relationship between the EQ-5D-5L sum and POD or FTL, we performed another OLS regression, which did not show significance concerning the main predictors (POD: β = −0.13 [−0.75, 0.49], *p* = 0.672; FTL: β = 0.07 [−0.11, 0.26], *p* = 0.630). Model assumptions and multicollinearity were of no concern in this model either. Again, some of the control variables showed positive effects, such as the number of medications and neurocognitive disease. This does not support the hypothesis, i.e., no association between POD or FTL and the EQ-5D-5L sum.

To test the association between the Barthel index and POD or FTL, we performed an OLS regression analysis using the same control variables. Again, the assumptions of this model were fulfilled, and multicollinearity was low. Another time, we could not find any significant effects of POD or FTL on BI (POD: β = 0.03 [−0.52, 0.58], *p* = 0.911; FTL: β = 0.02 [−0.15, 0.18], *p* = 0.911). However, some variables highlight an effect on BI, such as age, which has a positive effect. A negative effect onBI showed the number of medications, the EQ-5D-5L, and neurodegenerative disease. The results did not support our main hypothesis.

Finally, we performed another OLS regression to test the relationship between POD or FTL and the IQCODE. As with the previous regressions, the model assumptions and multicollinearity were of no concern, and there were no significant results regarding the association between POD or FTL and IQCODE (POD: β = −0.10 [−0.89, 0.69], *p* = 0.964; FTL: β = −0.15 [−0.38, 0.09], *p* = 0.593). In this model, the MoCA test as a control variable showed a negative relationship with the IQCODE. The hypothesis that there is an association between POD or FTL and the IQCODE is not supported.

Sensitivity Analysis: Our descriptive analyses suggested that rates of missing values on the SBT, FTL, GDS, and MoCA variables were not negligible (>5%). To assess the robustness of our findings to this missingness, we imputed missing values on these variables using a predictive mean matching approach and re-estimated our four main models on this imputed data [32]. While this suggested changes to the effects of some of our statistical controls, the results regarding FTL appeared to be widely robust. The effects of POD were slightly more positive for the imputed data but did not reach statistical significance for any of our four outcomes of interest.

### 3.3. Exploratory Analysis

Our exploratory analysis revealed a few isolated associations, mainly involving IADL, but overall, neither the additional preoperative variables nor the main factors POD and FTL showed significant links, including mortality (Table A8).

## 4. Discussion

This section examines the potential impact of postoperative delirium and liberal fluid fasting on long-term cognitive and functional outcomes in elderly trauma patients twelve months after surgery, with a brief overview of each test included in the analysis.

Firstly, the relationship between the Short Blessed Test and postoperative delirium, as well as fasting time liquids, has not been confirmed. Although the SBT showed no significant association with POD or FTL, prior studies have identified delirium as a strong predictor of those able to complete testing. Still, descriptive data show noticeable differences in SBT scores between patients with and without POD, indicating a potential, though nonsignificant, association. Fasting time for liquids also showed no correlation with SBT performance, which is unsurprising given the 12-month session; neurodegenerative disease emerged as a significant predictor in all tests except the IQCODE, supporting the expectation that affected patients show greater cognitive impairment and reduced functional independence. Given their elevated baseline risk for delirium, due to the disease itself or delirium-provoking medications, a poorer long-term outcome in this group is plausible [33,34]. Neurocognitive disorders are characterised by impairments in cognitive functions such as memory, attention, language and executive functioning, which are caused by underlying brain pathology.

In this model, the Charlson Comorbidity Index, MoCA, and EQ-5D-5L also reached significance. Higher comorbidity and lower MoCA scores were associated with poorer SBT performance, consistent with evidence that mild cognitive impairment increases delirium risk and contributes to subsequent cognitive decline [33].

The EQ-5D-5L sum score showed no significant association with POD or FTL. Although the regression suggests no effect of POD on quality of life, the descriptive data indicate poorer scores in patients who experienced delirium.

Two additional predictors of the EQ-5D-5L were medication count and neurodegenerative disease. Notably, medication count showed a strong association, whereas the Charlson Comorbidity Index did not. Although one might expect polypharmacy to reflect a higher comorbidity burden, it is independently linked to functional and cognitive decline, which supports our findings [35,36].

In general, studies have shown that POD affects quality of life and leads to functional decline in the long term, although our tests could not prove this [37].

A limitation of the EQ-5D-5L analysis is that we used the sum score rather than the recommended index. Due to structural barriers complicating index calculation, we retained both the sum and baseline values to allow for better comparison.

The Barthel index showed a similar pattern as the EQ-5D-5L, with no support for the main hypotheses. Descriptive data suggest a correlation, as non-delirium patients had higher mean scores. Significant predictors in the regression included medication count and neurodegenerative disease, likely affecting self-care and daily living. Associations with age and EQ-5D-5L may reflect age-related functional decline or overlapping test items.

The IQCODE did not show an association with POD or FTL. It was correlated with MoCA, reflecting pre-existing cognitive impairment and worse outcomes at 12 months. However, the model’s predictive value was limited (low adjusted R-squared). Although the test is recommended to be completed by a close relative, this was not always possible. The literature suggests a link between POD and higher IQCODE scores, supporting a potential relationship between delirium and cognitive decline [38]. Overall, the accuracy of the selected tests for assessing current cognition and quality of life remains uncertain.

Exploratory analyses revealed correlations between preoperative and follow-up tests, mainly for those with similar items, likely reflecting collinearity. We also conducted a mortality analysis due to the high mortality rate of 16%. None of the mechanisms we considered showed any significant effect.

The observed association between polypharmacy and adverse long-term outcomes is consistent with previous research. Polypharmacy has been linked to cognitive impairment, functional decline, frailty, falls, and hospitalisation in older adults. Given the high prevalence of multimorbidity in geriatric populations, the burden of medication may represent an important and potentially modifiable risk factor for adverse cognitive and functional trajectories [39].

The strong association between pre-existing neurocognitive disease and poorer long-term outcomes is biologically plausible. Individuals with underlying cognitive impairment may have reduced cognitive reserve, making them more vulnerable to further cognitive and functional decline following acute health stressors. This finding highlights the importance of identifying cognitive impairment in older surgical patients early on and supports the need for targeted perioperative management strategies. The current literature also supports the finding that Niu et al. identified preoperative cognitive impairment as a significant risk factor for the development of postoperative delirium [40].

Current evidence suggests that postoperative delirium should be considered not only an acute perioperative complication but also a marker of underlying brain vulnerability reflecting the interaction of pre-existing cognitive reserve, biological susceptibility and perioperative stressors [41].

In summary, our findings did not support the hypothesised influence of POD or FTL on SBT, EQ-5D-5L, BI, or IQCODE. Although postoperative delirium has consistently been associated with adverse long-term outcomes in previous studies, no significant associations were observed in the present analysis. However, descriptive and regression analyses indicated associations that warrant further investigation. The absence of effects of the small intervention after one year suggests that the approach may primarily yield short-term benefits. Although there were no significant associations between POD, fluid fasting duration and long-term outcomes, the findings have potential clinical implications. The results emphasise the importance of a comprehensive geriatric assessment, including identifying pre-existing neurocognitive disease and polypharmacy, as these factors emerged as relevant predictors of long-term outcomes. Routine delirium screening remains important, as POD is consistently associated with adverse outcomes in the broader literature and may contribute to long-term vulnerability in older patients.

Nevertheless, the LFFgertrud follow-up study has several strengths: it is longitudinal, with a second assessment twelve months after baseline, allowing more reliable results and better visualisation of changes [42]. Additionally, sensitivity analyses demonstrate the robustness of the findings. A particular strength of the present study is the use of the 4AT for delirium screening at baseline. The 4AT is a well-established and validated screening instrument, and recent evidence from the validation of versions in other languages further supports its reliability and diagnostic accuracy across different linguistic and cultural settings [13].

Several limitations affect this study. Unknown confounders over the 12-month follow-up, differing baseline and follow-up tests, and additional hospitalisations or illnesses make it difficult to attribute changes in cognition or quality of life to single factors such as POD or liberal fluid fasting. Although delirium is linked to long-term cognitive decline in the literature [9], this was not evident in our results, likely reflecting multifactorial influences. Cognitive outcomes were only available among survivors, potentially introducing survivor bias. The study included only non-randomised trauma surgery patients, with more non-delirious than delirious participants, introducing potential selection bias. As in many longitudinal studies, the sample may overrepresent motivated, healthier individuals rather than the general population [42].

Furthermore, our methods need critical evaluation. Only four conceptually similar tests were used, raising the risk of multicollinearity. Telephone-based assessments require simplified tasks and may have limited test complexity, which could affect accuracy. Furthermore, the absence of face-to-face evaluation and inconsistent completion of the IQCODE by informants may have introduced measurement bias. Challenges included variable participant motivation, possible cheating, mid-interview dropouts, and difficulty contacting participants. All surveys were conducted by a single assessor, reducing variability but potentially introducing subjective bias. However, the prospective approach and low loss-to-follow-up rate might partially offset these shortcomings. Another limitation is the relatively small sample size. Although a sensitivity analysis was performed, the study may have lacked the statistical power required to detect small-to-moderate effects. Therefore, the absence of statistically significant associations should be interpreted with caution, as this could reflect either a true lack of association or insufficient statistical power. Additionally, health events that occurred during the follow-up period and were not measured may have influenced the observed long-term outcomes.

Future research should use more comprehensive assessments at multiple time points to better investigate the effects of POD or FTL on quality of life and cognitive decline.

Preventing postoperative delirium remains a key strategy to preserve cognitive and functional outcomes in older surgical patients. The present findings support the need for further research into perioperative strategies, such as optimised preoperative fluid management, to reduce the risk of delirium.

## 5. Conclusions

In conclusion, our findings did not provide evidence of a significant association between postoperative delirium, the duration of fluid fasting, and cognitive, functional, or quality-of-life outcomes one year after hospitalisation. However, pre-existing neurocognitive impairment and polypharmacy were associated with poorer long-term outcomes. This highlights the importance of underlying patient characteristics in this vulnerable population. Due to the limited statistical power resulting from loss to follow-up, these findings should be interpreted with caution. Further research involving larger sample sizes and higher retention rates is required to confirm these results and identify modifiable factors that could influence long-term recovery.

## Figures and Tables

**Table 1:** Summary table of demographics.

	Conventional Fasting (*N* = 39)	Liberal Fluid Fasting (*N* = 50)	Overall (*N* = 89)
Sex			
Female	26 (66.7%)	23 (46.0%)	49 (55.1%)
Male	13 (33.3%)	27 (54.0%)	40 (44.9%)
Age (Years)			
Mean (SD)	79.2 (5.21)	79.4 (5.68)	79.3 (5.45)
Median [Min, Max]	79.0 [70.0, 91.0]	81.5 [69.0, 94.0]	80.0 [69.0, 94.0]
Weight (kg)			
Mean (SD)	72.7 (16.0)	79.0 (17.7)	76.2 (17.2)
Median [Min, Max]	73.0 [47.0, 124]	75.0 [50.0, 124]	73.0 [47.0, 124]
Height (cm)			
Mean (SD)	167 (9.02)	171 (8.95)	169 (9.17)
Median [Min, Max]	167 [154, 186]	171 [156, 193]	168 [154, 193]
Charlson Comorbidity Index			
Mean (SD)	1.34 (1.82)	1.78 (1.88)	1.59 (1.85)
Median [Min, Max]	1.00 [0, 9.00]	1.50 [0, 6.00]	1.00 [0, 9.00]
Missing	1 (2.6%)	0 (0%)	1 (1.1%)
Medications			
Mean (SD)	7.72 (4.93)	8.32 (5.79)	8.06 (5.41)
Median [Min, Max]	7.00 [1.00, 22.0]	8.50 [0, 18.0]	7.00 [0, 22.0]

**Table 2:** Summary table of follow-up outcome variables sorted by fasting regime and delirium.

	Conventional Fasting		Liberal Fluid Fasting		Overall	
	No POD (*N* = 29)	POD (*N* = 10)	No POD (*N* = 43)	POD (*N* = 7)	No POD (*N* = 72)	POD (*N* = 17)
**SBT**						
Mean (SD)	5.12 (4.31)	6.29 (5.82)	5.24 (4.64)	8.67 (2.42)	5.19 (4.48)	7.38 (4.57)
Median [Min, Max]	3.00 [0, 14.0]	4.00 [0, 14.0]	4.00 [0, 18.0]	10.0 [4.00, 10.0]	4.00 [0, 18.0]	8.00 [0, 14.0]
Missing	3 (10.3%)	3 (30.0%)	2 (4.7%)	1 (14.3%)	5 (6.9%)	4 (23.5%)
**Sum**						
Mean (SD)	10.2 (4.32)	11.0 (5.10)	9.63 (4.17)	15.9 (2.73)	9.85 (4.21)	13.3 (4.74)
Median [Min, Max]	9.00 [6.00, 20.0]	9.00 [6.00, 20.0]	9.00 [5.00, 21.0]	15.0 [11.0, 19.0]	9.00 [5.00, 21.0]	15.0 [6.00, 20.0]
Missing	1 (3.4%)	2 (20.0%)	0 (0%)	0 (0%)	1 (1.4%)	2 (11.8%)
**Mob**						
Mean (SD)	2.29 (1.30)	2.50 (1.41)	2.21 (1.15)	4.14 (0.690)	2.24 (1.20)	3.27 (1.39)
Median [Min, Max]	2.00 [1.00, 5.00]	2.00 [1.00, 5.00]	2.00 [1.00, 5.00]	4.00 [3.00, 5.00]	2.00 [1.00, 5.00]	4.00 [1.00, 5.00]
Missing	1 (3.4%)	2 (20.0%)	0 (0%)	0 (0%)	1 (1.4%)	2 (11.8%)
**Sc**						
Mean (SD)	1.75 (1.14)	1.75 (1.16)	1.63 (1.07)	3.71 (1.25)	1.68 (1.09)	2.67 (1.54)
Median [Min, Max]	1.00 [1.00, 4.00]	1.00 [1.00, 4.00]	1.00 [1.00, 5.00]	4.00 [1.00, 5.00]	1.00 [1.00, 5.00]	3.00 [1.00, 5.00]
Missing	1 (3.4%)	2 (20.0%)	0 (0%)	0 (0%)	1 (1.4%)	2 (11.8%)
**UA**						
Mean (SD)	2.04 (1.32)	2.25 (1.49)	1.91 (1.11)	4.00 (1.00)	1.96 (1.19)	3.07 (1.53)
Median [Min, Max]	1.00 [1.00, 5.00]	1.50 [1.00, 4.00]	1.00 [1.00, 5.00]	4.00 [2.00, 5.00]	1.00 [1.00, 5.00]	4.00 [1.00, 5.00]
Missing	1 (3.4%)	2 (20.0%)	0 (0%)	0 (0%)	1 (1.4%)	2 (11.8%)
**PD**						
Mean (SD)	2.46 (0.793)	2.88 (0.835)	2.14 (0.889)	2.14 (0.378)	2.27 (0.861)	2.53 (0.743)
Median [Min, Max]	2.00 [1.00, 4.00]	3.00 [2.00, 4.00]	2.00 [1.00, 4.00]	2.00 [2.00, 3.00]	2.00 [1.00, 4.00]	2.00 [2.00, 4.00]
Missing	1 (3.4%)	2 (20.0%)	0 (0%)	0 (0%)	1 (1.4%)	2 (11.8%)
**AD**						
Mean (SD)	1.64 (0.951)	1.63 (1.06)	1.74 (0.978)	1.86 (0.900)	1.70 (0.962)	1.73 (0.961)
Median [Min, Max]	1.00 [1.00, 4.00]	1.00 [1.00, 4.00]	1.00 [1.00, 4.00]	2.00 [1.00, 3.00]	1.00 [1.00, 4.00]	1.00 [1.00, 4.00]
Missing	1 (3.4%)	2 (20.0%)	0 (0%)	0 (0%)	1 (1.4%)	2 (11.8%)
**BI**						
Mean (SD)	84.5 (25.2)	83.8 (24.9)	89.1 (18.2)	45.7 (15.9)	87.3 (21.2)	66.0 (28.4)
Median [Min, Max]	97.5 [25.0, 100]	97.5 [30.0, 100]	100 [30.0, 100]	45.0 [35.0, 80.0]	100 [25.0, 100]	70.0 [30.0, 100]
Missing	1 (3.4%)	2 (20.0%)	0 (0%)	0 (0%)	1 (1.4%)	2 (11.8%)
**IQCODEs**						
Mean (SD)	3.23 (0.378)	3.36 (0.495)	3.19 (0.320)	3.14 (0.247)	3.21 (0.342)	3.26 (0.401)
Median [Min, Max]	3.00 [3.00, 4.00]	3.00 [3.00, 4.00]	3.00 [2.71, 4.43]	3.00 [3.00, 3.57]	3.00 [2.71, 4.43]	3.00 [3.00, 4.00]
Missing	1 (3.4%)	2 (20.0%)	0 (0%)	0 (0%)	1 (1.4%)	2 (11.8%)
**dec**						
No	29 (100%)	10 (100%)	43 (100%)	7 (100%)	72 (100%)	17 (100%)
Yes	0 (0%)	0 (0%)	0 (0%)	0 (0%)	0 (0%)	0 (0%)
**rej**						
No	29 (100%)	10 (100%)	43 (100%)	7 (100%)	72 (100%)	17 (100%)
Yes	0 (0%)	0 (0%)	0 (0%)	0 (0%)	0 (0%)	0 (0%)
**nr**						
No	29 (100%)	10 (100%)	43 (100%)	7 (100%)	72 (100%)	17 (100%)
Yes	0 (0%)	0 (0%)	0 (0%)	0 (0%)	0 (0%)	0 (0%)

Note. Sum: EQ-5D-5L sum; Mob: mobility; Sc: self-care; UA: usual activities; PD: pain/discomfort; AD: anxiety/depression; dec: deceased; rej: rejected; nr: not reachable.

**Table 3:** Effect of demographic, selected baseline and follow-up variables on SBT.

Effect	Short Blessed Test			
	Std. Beta	Standardized Std. Error	Statistic	*p*
(Intercept)	0.10 (−0.20–0.40)	0.15	0.52	0.736
Fasting Time Liquids	−0.00 (−0.22–0.21)	0.11	−0.03	0.980
Postoperative Delirium	−0.52 (−1.25–0.20)	0.36	−1.44	0.285
Sex (male)	−0.20 (−0.63–0.23)	0.21	−0.94	0.551
Age	0.08 (−0.14–0.30)	0.11	0.71	0.662
CCI	0.27 (0.05–0.48)	0.11	2.47	0.091
Number of Medications	0.03 (−0.22–0.29)	0.13	0.26	0.873
MoCA	−0.23 (−0.45–−0.02)	0.11	−2.17	0.101
EQ-5D-5L	0.25 (0.02–0.48)	0.12	2.13	0.101
Neurodegenerative Disease (Yes)	1.27 (0.32–2.22)	0.48	2.67	0.091
Depression (Yes)	−0.68 (−1.61–0.24)	0.46	−1.48	0.285
R^2^/R^2^ adjusted	0.350/0.243			

Note. To correct for multiple comparisons, the *p*-values have been adjusted using the Benjamini–Hochberg false discovery rate correction [29].

**Table 4:** Effect of demographic, selected baseline and follow-up variables on EQ-5D-5L sum.

Effect	EQ-5D-5L (Sum Score)			
	Std. Beta	Standardized Std. Error	Statistic	*p*
(Intercept)	−0.25 (−0.51–0.02)	0.13	1.62	0.300
Fasting Time Liquids	0.07 (−0.11–0.26)	0.09	0.81	0.630
Postoperative Delirium	−0.13 (−0.75–0.49)	0.31	−0.43	0.672
Sex (male)	0.21 (−0.16–0.58)	0.19	1.12	0.585
Age	−0.06 (−0.25–0.13)	0.10	−0.64	0.630
CCI	0.09 (−0.10–0.27)	0.09	0.95	0.630
Number of Medications	0.30 * (0.09–0.52)	0.11	2.81	0.036
MoCA	−0.07 (−0.26–0.12)	0.09	−0.74	0.630
EQ-5D-5L	0.19 (−0.00–0.39)	0.10	1.95	0.203
Neurodegenerative Disease (Yes)	1.43 ** (0.69–2.18)	0.37	3.84	0.003
Depression (Yes)	0.23 (−0.58–1.05)	0.41	0.57	0.630
R^2^/R^2^ adjusted	0.488/0.408			

Note. To correct for multiple comparisons, the *p*-values have been adjusted using the Benjamini-Hochberg false discovery rate correction [29]. * *p* < 0.05 ** *p* < 0.01.

**Table 5:** Effect of demographic, selected baseline and follow-up variables on the Barthel index.

Effect	Barthel Index			
	Std. Beta	Standardized Std. Error	Statistic	*p*
(Intercept)	0.17 (−0.07–0.40)	0.12	1.17	0.450
Fasting Time Liquids	0.02 (−0.15–0.18)	0.08	0.22	0.911
Postoperative Delirium	0.03 (−0.52–0.58)	0.28	0.11	0.911
Sex (male)	−0.02 (−0.35–0.31)	0.17	−0.13	0.911
Age	0.18 (0.01–0.35)	0.09	2.13	0.102
CCI	−0.09 (−0.25–0.08)	0.08	−1.03	0.480
Number of Medications	−0.27 * (−0.46–−0.07)	0.10	−2.77	0.041
MoCA	0.16 (−0.01–0.33)	0.08	1.91	0.134
EQ-5D-5L	−0.22 (−0.40–−0.05)	0.09	−2.52	0.052
Neurodegenerative Disease (Yes)	−1.63 *** (−2.29–−0.96)	0.33	−4.88	<0.001
Depression (Yes)	0.23 (−0.50–0.96)	0.36	0.63	0.729
R^2^/R^2^ adjusted	0.593/0.529			

Note. To correct for multiple comparisons, the *p*-values have been adjusted using the Benjamini-Hochberg false discovery rate correction [29]. * *p* < 0.05 *** *p* < 0.001.

**Table 6:** Effect of demographic, selected baseline and follow-up variables on IQCODEs.

Effect	IQCODE Score			
	Std. Beta	Standardized Std. Error	Statistic	*p*
(Intercept)	−0.09 (−0.43–0.25)	0.17	4.21	0.001
Fasting Time Liquids	−0.15 (−0.38–0.09)	0.12	−1.25	0.593
Postoperative Delirium	−0.10 (−0.89–0.69)	0.39	−0.25	0.964
Sex (male)	0.06 (−0.41–0.53)	0.24	0.26	0.964
Age	0.22 (−0.03–0.46)	0.12	1.77	0.297
CCI	0.01 (−0.23–0.24)	0.12	0.05	0.964
Number of Medications	−0.03 (−0.31–0.24)	0.14	−0.23	0.964
MoCA	−0.24 (−0.48–−0.00)	0.12	−2.01	0.265
EQ-5D-5L	0.02 (−0.24–0.27)	0.13	0.12	0.964
Neurodegenerative Disease (Yes)	0.47 (−0.47–1.42)	0.47	1.00	0.612
Depression (Yes)	0.50 (−0.53–1.54)	0.52	0.97	0.612
R^2^/R^2^ adjusted	0.175/0.046			

Note. To correct for multiple comparisons, the *p*-values have been adjusted using the Benjamini-Hochberg false discovery rate correction [29].

## Data Availability

The raw data supporting the conclusions of this article will be made available by the authors on request.

## Abbreviations

The following abbreviations are used in this manuscript:

POD	Postoperative Delirium
LFF	Liberal Fluid Fasting
FTL	Fasting Time Liquids
SBT	Short Blessed Test
EQ-5D-5L	European Quality of Life 5 Dimensions 5 Level Version
IQCODE	Informant Questionnaire on Cognitive Decline in the Elderly
BI	Barthel Index
MoCA	Montreal Cognitive Assessment
Sum	Sum of EQ-5D-5L
Mob	Mobility
Sc	Self-Care
UA	Usual Activities
PD	Pain/Discomfort
AD	Anxiety/Depression
CCI	Charlson Comorbidity Index

## Appendix A

**Table A1:** Overview of assessment tools.

Instrument	Construct Assessed	Structure and Scoring	Interpretation
Short Blessed Test (SBT) [25]	Cognitive function (orientation, memory, concentration)	6 items; total score 0–28	0–8: normal–minimal impairment; 9–19: minimal–moderate; 20–28: severe impairment
EQ-5D-5L [28]	Health-related quality of life	5 dimensions (mobility, self-care, usual activities, pain/discomfort, anxiety/depression); 5 severity levels	Higher response levels reflect greater impairment; produces descriptive health profile
IQCODE [26,43]	Informant-rated cognitive decline	7 items, scored 1–5 relative to function 2 years prior; total score > 23 or mean > 3.29	Higher scores indicate greater decline
Barthel Index [27]	Physical function and independence in activities of daily living	10 items; total score 0–100	Higher scores indicate greater independence

Note: Proxy ratings were used for five patients for the Barthel index, EQ-5D-5L, and IQCODE when direct patient assessment was not possible.

**Table A2:** Summary table of baseline LFF.

	Conservative Fasting (*N* = 39)	Liberal Fluid Fasting (*N* = 50)	Overall (*N* = 89)
Fasting Time Liquids (h)			
Mean (SD)	7.72 (5.33)	1.89 (1.96)	4.41 (4.76)
Median [Min, Max]	5.50 [1.00, 16.0]	1.00 [0, 7.00]	2.50 [0, 16.0]
Missing	4 (10.3%)	4 (8.0%)	8 (9.0%)
MoCA			
Mean (SD)	23.4 (5.31)	23.0 (4.69)	23.1 (4.93)
Median [Min, Max]	25.0 [8.00, 30.0]	23.5 [9.00, 30.0]	24.0 [8.00, 30.0]
Missing	5 (12.8%)	0 (0%)	5 (5.6%)
FRAIL scale			
Mean (SD)	1.46 (1.23)	1.33 (1.29)	1.39 (1.26)
Median [Min, Max]	2.00 [0, 4.00]	1.00 [0, 4.00]	2.00 [0, 4.00]
Missing	0 (0%)	2 (4.0%)	2 (2.2%)
MNALF			
Mean (SD)	23.4 (3.44)	25.1 (3.44)	24.4 (3.53)
Median [Min, Max]	24.3 [15.0, 28.0]	26.0 [10.0, 29.0]	25.0 [10.0, 29.0]
Missing	1 (2.6%)	2 (4.0%)	3 (3.4%)
EQ5D5L			
Mean (SD)	10.6 (5.45)	10.5 (4.24)	10.6 (4.79)
Median [Min, Max]	9.00 [5.00, 22.0]	10.0 [3.00, 19.0]	9.00 [3.00, 22.0]
Missing	0 (0%)	2 (4.0%)	2 (2.2%)
Mobility			
Mean (SD)	2.61 (1.42)	2.74 (1.37)	2.69 (1.38)
Median [Min, Max]	2.00 [1.00, 5.00]	3.00 [1.00, 5.00]	3.00 [1.00, 5.00]
Missing	11 (28.2%)	11 (22.0%)	22 (24.7%)
Selfcare			
Mean (SD)	2.11 (1.34)	1.97 (1.18)	2.03 (1.24)
Median [Min, Max]	2.00 [1.00, 5.00]	2.00 [1.00, 5.00]	2.00 [1.00, 5.00]
Missing	11 (28.2%)	11 (22.0%)	22 (24.7%)
Activities			
Mean (SD)	2.43 (1.50)	1.95 (1.23)	2.15 (1.36)
Median [Min, Max]	2.00 [1.00, 5.00]	1.00 [1.00, 5.00]	2.00 [1.00, 5.00]
Missing	11 (28.2%)	11 (22.0%)	22 (24.7%)
Pain			
Mean (SD)	2.21 (1.20)	1.97 (0.986)	2.07 (1.08)
Median [Min, Max]	2.00 [1.00, 5.00]	2.00 [1.00, 4.00]	2.00 [1.00, 5.00]
Missing	11 (28.2%)	11 (22.0%)	22 (24.7%)
Depression			
Mean (SD)	1.39 (0.875)	1.56 (0.882)	1.49 (0.877)
Median [Min, Max]	1.00 [1.00, 5.00]	1.00 [1.00, 4.00]	1.00 [1.00, 5.00]
Missing	11 (28.2%)	11 (22.0%)	22 (24.7%)
IADL			
Mean (SD)	6.33 (2.46)	6.27 (2.69)	6.30 (2.57)
Median [Min, Max]	8.00 [0, 8.00]	8.00 [0, 8.00]	8.00 [0, 8.00]
Missing	0 (0%)	2 (4.0%)	2 (2.2%)
GDS			
Mean (SD)	1.70 (2.60)	1.62 (2.57)	1.65 (2.57)
Median [Min, Max]	1.00 [0, 11.0]	1.00 [0, 12.0]	1.00 [0, 12.0]
Missing	2 (5.1%)	3 (6.0%)	5 (5.6%)
Albumin			
Mean (SD)	3.35 (0.602)	3.12 (0.527)	3.26 (0.571)
Median [Min, Max]	3.20 [2.40, 4.20]	3.00 [2.60, 4.10]	3.10 [2.40, 4.20]
Missing	28 (71.8%)	44 (88.0%)	72 (80.9%)
GFR			
Mean (SD)	72.6 (19.3)	72.5 (19.2)	72.5 (19.1)
Median [Min, Max]	81.0 [28.0, 108]	79.0 [11.0, 96.0]	80.0 [11.0, 108]
Missing	0 (0%)	3 (6.0%)	3 (3.4%)
CRP			
Mean (SD)	2.43 (3.35)	4.69 (6.82)	3.71 (5.66)
Median [Min, Max]	1.10 [0.100, 13.5]	2.40 [0.100, 29.9]	1.90 [0.100, 29.9]
Missing	1 (2.6%)	1 (2.0%)	2 (2.2%)
Surgical Risk			
1	19 (48.7%)	41 (82.0%)	60 (67.4%)
2	19 (48.7%)	9 (18.0%)	28 (31.5%)
Missing	1 (2.6%)	0 (0%)	1 (1.1%)
Duration of Anaesthesia			
Mean (SD)	194 (79.2)	171 (93.0)	181 (87.5)
Median [Min, Max]	191 [72.0, 476]	164 [55.0, 568]	174 [55.0, 568]
Duration of Operation			
Mean (SD)	113 (59.7)	95.8 (74.1)	103 (68.3)
Median [Min, Max]	100 [20.0, 270]	91.0 [10.0, 445]	94.0 [10.0, 445]
Type of Anaesthesia			
Analgo	0 (0%)	0 (0%)	0 (0%)
Combined	10 (25.6%)	18 (36.0%)	28 (31.5%)
General	22 (56.4%)	29 (58.0%)	51 (57.3%)
Regional	7 (17.9%)	3 (6.0%)	10 (11.2%)
Hypotension			
Mean (SD)	7.41 (9.47)	6.86 (9.63)	7.10 (9.51)
Median [Min, Max]	3.00 [0, 35.0]	3.00 [0, 42.0]	3.00 [0, 42.0]
Infusion			
Mean (SD)	92.1 (210)	45.0 (204)	65.3 (207)
Median [Min, Max]	0 [0, 1000]	0 [0, 1050]	0 [0, 1050]
Missing	1 (2.6%)	0 (0%)	1 (1.1%)

**Table A3:** Summary table: Baseline delirium.

	Delirium No (*N* = 72)	Delirium Yes (*N* = 17)	Overall (*N* = 89)
Fasting Time Liquids (h)			
Mean (SD)	4.34 (4.68)	4.79 (5.34)	4.41 (4.76)
Median [Min, Max]	2.25 [0.250, 16.0]	3.50 [0, 16.0]	2.50 [0, 16.0]
Missing	4 (5.6%)	4 (23.5%)	8 (9.0%)
MoCA			
Mean (SD)	23.2 (5.03)	22.9 (4.52)	23.1 (4.93)
Median [Min, Max]	24.0 [8.00, 30.0]	23.5 [15.0, 30.0]	24.0 [8.00, 30.0]
Missing	2 (2.8%)	3 (17.6%)	5 (5.6%)
FRAIL scale			
Mean (SD)	1.30 (1.22)	1.76 (1.39)	1.39 (1.26)
Median [Min, Max]	1.00 [0, 4.00]	2.00 [0, 4.00]	2.00 [0, 4.00]
Missing	2 (2.8%)	0 (0%)	2 (2.2%)
MNALF			
Mean (SD)	25.0 (2.73)	21.8 (5.08)	24.4 (3.53)
Median [Min, Max]	25.5 [17.0, 29.0]	22.0 [10.0, 28.0]	25.0 [10.0, 29.0]
Missing	3 (4.2%)	0 (0%)	3 (3.4%)
EQ5D5L			
Mean (SD)	9.69 (4.20)	14.3 (5.39)	10.6 (4.79)
Median [Min, Max]	9.00 [3.00, 22.0]	16.0 [5.00, 22.0]	9.00 [3.00, 22.0]
Missing	2 (2.8%)	0 (0%)	2 (2.2%)
Mobility			
Mean (SD)	2.55 (1.30)	3.33 (1.61)	2.69 (1.38)
Median [Min, Max]	2.00 [1.00, 5.00]	3.50 [1.00, 5.00]	3.00 [1.00, 5.00]
Missing	17 (23.6%)	5 (29.4%)	22 (24.7%)
Selfcare			
Mean (SD)	1.82 (1.07)	3.00 (1.54)	2.03 (1.24)
Median [Min, Max]	1.00 [1.00, 5.00]	2.50 [1.00, 5.00]	2.00 [1.00, 5.00]
Missing	17 (23.6%)	5 (29.4%)	22 (24.7%)
Activities			
Mean (SD)	1.87 (1.12)	3.42 (1.68)	2.15 (1.36)
Median [Min, Max]	1.00 [1.00, 5.00]	3.50 [1.00, 5.00]	2.00 [1.00, 5.00]
Missing	17 (23.6%)	5 (29.4%)	22 (24.7%)
Pain			
Mean (SD)	2.07 (1.05)	2.08 (1.24)	2.07 (1.08)
Median [Min, Max]	2.00 [1.00, 4.00]	2.00 [1.00, 5.00]	2.00 [1.00, 5.00]
Missing	17 (23.6%)	5 (29.4%)	22 (24.7%)
Depression			
Mean (SD)	1.42 (0.875)	1.83 (0.835)	1.49 (0.877)
Median [Min, Max]	1.00 [1.00, 5.00]	2.00 [1.00, 3.00]	1.00 [1.00, 5.00]
Missing	17 (23.6%)	5 (29.4%)	22 (24.7%)
IADL			
Mean (SD)	6.93 (1.91)	3.71 (3.31)	6.30 (2.57)
Median [Min, Max]	8.00 [0, 8.00]	3.00 [0, 8.00]	8.00 [0, 8.00]
Missing	2 (2.8%)	0 (0%)	2 (2.2%)
GDS			
Mean (SD)	1.59 (2.59)	1.94 (2.52)	1.65 (2.57)
Median [Min, Max]	0 [0, 12.0]	2.00 [0, 10.0]	1.00 [0, 12.0]
Missing	4 (5.6%)	1 (5.9%)	5 (5.6%)
Albumin			
Mean (SD)	3.24 (0.553)	3.32 (0.676)	3.26 (0.571)
Median [Min, Max]	3.10 [2.60, 4.10]	3.20 [2.40, 4.20]	3.10 [2.40, 4.20]
Missing	60 (83.3%)	12 (70.6%)	72 (80.9%)
GFR			
Mean (SD)	72.7 (18.5)	71.7 (21.9)	72.5 (19.1)
Median [Min, Max]	81.0 [11.0, 98.0]	79.0 [28.0, 108]	80.0 [11.0, 108]
Missing	3 (4.2%)	0 (0%)	3 (3.4%)
CRP			
Mean (SD)	3.23 (4.65)	5.65 (8.60)	3.71 (5.66)
Median [Min, Max]	2.30 [0.100, 29.9]	1.80 [0.100, 29.9]	1.90 [0.100, 29.9]
Missing	2 (2.8%)	0 (0%)	2 (2.2%)
Surgical Risk			
1	51 (70.8%)	9 (52.9%)	60 (67.4%)
2	20 (27.8%)	8 (47.1%)	28 (31.5%)
Missing	1 (1.4%)	0 (0%)	1 (1.1%)
Duration of Anaesthesia			
Mean (SD)	181 (91.1)	182 (73.0)	181 (87.5)
Median [Min, Max]	168 [55.0, 568]	194 [60.0, 297]	174 [55.0, 568]
Duration of Operation			
Mean (SD)	103 (70.2)	105 (61.6)	103 (68.3)
Median [Min, Max]	87.0 [10.0, 445]	118 [10.0, 175]	94.0 [10.0, 445]
Type of Anaesthesia			
Analgo	0 (0%)	0 (0%)	0 (0%)
Combined	26 (36.1%)	2 (11.8%)	28 (31.5%)
General	39 (54.2%)	12 (70.6%)	51 (57.3%)
Regional	7 (9.7%)	3 (17.6%)	10 (11.2%)
Hypotension			
Mean (SD)	6.54 (9.27)	9.47 (10.4)	7.10 (9.51)
Median [Min, Max]	3.00 [0, 42.0]	6.00 [0, 35.0]	3.00 [0, 42.0]
Infusion			
Mean (SD)	66.9 (226)	58.8 (93.9)	65.3 (207)
Median [Min, Max]	0 [0, 1050]	0 [0, 200]	0 [0, 1050]
Missing	1 (1.4%)	0 (0%)	1 (1.1%)

**Table A4:** Summary table: Follow-up outcome variables against delirium (no/yes).

	No (*N* = 72)	Yes (*N* = 17)	Overall (*N* = 89)
SBT			
Mean (SD)	5.19 (4.48)	7.38 (4.57)	5.55 (4.54)
Median [Min, Max]	4.00 [0, 18.0]	8.00 [0, 14.0]	4.00 [0, 18.0]
Missing	5 (6.9%)	4 (23.5%)	9 (10.1%)
Sum			
Mean (SD)	9.85 (4.21)	13.3 (4.74)	10.4 (4.47)
Median [Min, Max]	9.00 [5.00, 21.0]	15.0 [6.00, 20.0]	9.00 [5.00, 21.0]
Missing	1 (1.4%)	2 (11.8%)	3 (3.4%)
Mob			
Mean (SD)	2.24 (1.20)	3.27 (1.39)	2.42 (1.29)
Median [Min, Max]	2.00 [1.00, 5.00]	4.00 [1.00, 5.00]	2.00 [1.00, 5.00]
Missing	1 (1.4%)	2 (11.8%)	3 (3.4%)
Sc			
Mean (SD)	1.68 (1.09)	2.67 (1.54)	1.85 (1.23)
Median [Min, Max]	1.00 [1.00, 5.00]	3.00 [1.00, 5.00]	1.00 [1.00, 5.00]
Missing	1 (1.4%)	2 (11.8%)	3 (3.4%)
UA			
Mean (SD)	1.96 (1.19)	3.07 (1.53)	2.15 (1.32)
Median [Min, Max]	1.00 [1.00, 5.00]	4.00 [1.00, 5.00]	2.00 [1.00, 5.00]
Missing	1 (1.4%)	2 (11.8%)	3 (3.4%)
PD			
Mean (SD)	2.27 (0.861)	2.53 (0.743)	2.31 (0.844)
Median [Min, Max]	2.00 [1.00, 4.00]	2.00 [2.00, 4.00]	2.00 [1.00, 4.00]
Missing	1 (1.4%)	2 (11.8%)	3 (3.4%)
AD			
Mean (SD)	1.70 (0.962)	1.73 (0.961)	1.71 (0.956)
Median [Min, Max]	1.00 [1.00, 4.00]	1.00 [1.00, 4.00]	1.00 [1.00, 4.00]
Missing	1 (1.4%)	2 (11.8%)	3 (3.4%)
BI			
Mean (SD)	87.3 (21.2)	66.0 (28.4)	83.5 (23.8)
Median [Min, Max]	100 [25.0, 100]	70.0 [30.0, 100]	95.0 [25.0, 100]
Missing	1 (1.4%)	2 (11.8%)	3 (3.4%)
IQCODEs			
Mean (SD)	3.21 (0.342)	3.26 (0.401)	3.22 (0.351)
Median [Min, Max]	3.00 [2.71, 4.43]	3.00 [3.00, 4.00]	3.00 [2.71, 4.43]
Missing	1 (1.4%)	2 (11.8%)	3 (3.4%)

**Table A5:** Summary table: Follow-up outcome variables against conservative/LFF.

	Conservative (*N* = 39)	LFF (*N* = 50)	Overall (*N* = 89)
SBT			
Mean (SD)	5.36 (4.59)	5.68 (4.55)	5.55 (4.54)
Median [Min, Max]	4.00 [0, 14.0]	4.00 [0, 18.0]	4.00 [0, 18.0]
Missing	6 (15.4%)	3 (6.0%)	9 (10.1%)
Sum			
Mean (SD)	10.4 (4.44)	10.5 (4.54)	10.4 (4.47)
Median [Min, Max]	9.00 [6.00, 20.0]	9.50 [5.00, 21.0]	9.00 [5.00, 21.0]
Missing	3 (7.7%)	0 (0%)	3 (3.4%)
Mob			
Mean (SD)	2.33 (1.31)	2.48 (1.28)	2.42 (1.29)
Median [Min, Max]	2.00 [1.00, 5.00]	2.00 [1.00, 5.00]	2.00 [1.00, 5.00]
Missing	3 (7.7%)	0 (0%)	3 (3.4%)
Sc			
Mean (SD)	1.75 (1.13)	1.92 (1.31)	1.85 (1.23)
Median [Min, Max]	1.00 [1.00, 4.00]	1.00 [1.00, 5.00]	1.00 [1.00, 5.00]
Missing	3 (7.7%)	0 (0%)	3 (3.4%)
UA			
Mean (SD)	2.08 (1.34)	2.20 (1.31)	2.15 (1.32)
Median [Min, Max]	1.00 [1.00, 5.00]	2.00 [1.00, 5.00]	2.00 [1.00, 5.00]
Missing	3 (7.7%)	0 (0%)	3 (3.4%)
PD			
Mean (SD)	2.56 (0.809)	2.14 (0.833)	2.31 (0.844)
Median [Min, Max]	2.00 [1.00, 4.00]	2.00 [1.00, 4.00]	2.00 [1.00, 4.00]
Missing	3 (7.7%)	0 (0%)	3 (3.4%)
AD			
Mean (SD)	1.64 (0.961)	1.76 (0.960)	1.71 (0.956)
Median [Min, Max]	1.00 [1.00, 4.00]	1.00 [1.00, 4.00]	1.00 [1.00, 4.00]
Missing	3 (7.7%)	0 (0%)	3 (3.4%)
BI			
Mean (SD)	84.3 (24.8)	83.0 (23.4)	83.5 (23.8)
Median [Min, Max]	97.5 [25.0, 100]	95.0 [30.0, 100]	95.0 [25.0, 100]
Missing	3 (7.7%)	0 (0%)	3 (3.4%)
IQCODEs			
Mean (SD)	3.26 (0.402)	3.19 (0.309)	3.22 (0.351)
Median [Min, Max]	3.00 [3.00, 4.00]	3.00 [2.71, 4.43]	3.00 [2.71, 4.43]
Missing	3 (7.7%)	0 (0%)	3 (3.4%)

**Table A6:** Means, standard deviations, and correlations with confidence intervals.

Variable	*M*	*SD*	1	2	3	4
1. FTL	4.41	4.76				
2. SBT	5.55	4.54	−0.05			
			[−0.27, 0.18]			
3. Sum	10.44	4.47	−0.06	0.34 **		
			[−0.28, 0.16]	[0.13, 0.52]		
4. BI	83.55	23.84	0.10	−0.40 **	−0.90 **	
			[−0.12, 0.31]	[−0.57, −0.19]	[−0.93, −0.84]	
5. IQCODEs	3.22	0.35	−0.17	0.34 **	0.36 **	−0.35 **
			[−0.37, 0.06]	[0.13, 0.52]	[0.17, 0.54]	[−0.53, −0.15]

Note. *M* and *SD* are used to represent mean and standard deviation, respectively. Values in square brackets indicate the 95% confidence interval for each correlation. The confidence interval is a plausible range of population correlations that could have caused the sample correlation [44]. ** indicates *p* < 0.01.

### Background Details Exploratory Analysis

To provide a perspective for future studies, we explored associations between follow-up outcomes and additional preoperative variables, specifically IADL, the geriatric depression score, and the FRAIL scale (Table A7).

The regression with the SBT did not show any significant results. This was different with the EQ-5D-5L, where we saw a negative relationship with the IADL. These two tests use similar items, such as mobility and self-care, which could explain the substantial effect.

The FRAIL scale showed a less positive effect. The Barthel index also showed a positive association with the IADL, which could be explained in the same way as the relation between EQ-5D-5L and the IADL. The IQCODE had a small positive correlation with the FRAIL scale. This could also distort the model fit and explain the relatively high adjusted R-squared results in the models concerned.

In further analyses, we looked at mortality using regular regression (Table A8).

We took twelve baseline variables that might be related to the number of patients who died, such as CCI, medication, neurodegenerative disease, duration of anaesthesia and hypotension, to name a few. These were the predictor variables, and mortality was the outcome. The analysis showed no significance for any of the variables. Regarding the independent variables of our main hypothesis, POD (OR = 1.83 [0.40, 8.06], *p* = 0.422) and FTL (OR = 1.68 [0.94, 3.06], *p* = 0.079), we could not find any noticeable associations.

**Table A7:** Effect of further pre-OR cognitive tests on all follow-up tests.

Effect	Short Blessed Test			
	Std. Beta	Standardized Std. Error	Statistic	*p*
(Intercept)	0.00 (−0.22–0.22)	0.11	3.43	0.004
IADL	−0.16 (−0.40–0.08)	0.12	−1.35	0.241
Geriatric Depression Scale	0.23 (−0.02–0.49)	0.13	1.82	0.146
FRAIL Scale	0.03 (−0.22–0.28)	0.13	0.25	0.806
R^2^/R^2^ adjusted	0.114/0.077			
**Effect**	**EQ-5D-5L (Sum Score)**			
	**Std. Beta**	**Standardized Std. Error**	**Statistic**	***p***
(Intercept)	−0.00 (−0.18–0.18)	0.09	11.34	<0.001
IADL	−0.51 *** (−0.70–−0.33)	0.09	−5.52	<0.001
Geriatric Depression Scale	−0.08 (−0.28–0.12)	0.10	−0.83	0.410
FRAIL Scale	0.27 * (0.07–0.47)	0.10	2.72	0.011
R^2^/R^2^ adjusted	0.383/0.358			
**Effect**	**Barthel Index**			
	**Std. Beta**	**Standardized Std. Error**	**Statistic**	***p***
(Intercept)	−0.00 (−0.16–0.16)	0.08	7.39	<0.001
IADL	0.64 *** (0.47–0.81)	0.09	7.39	<0.001
Geriatric Depression Scale	−0.01 (−0.19–0.18)	0.09	−0.08	0.940
FRAIL Scale	−0.14 (−0.32–0.05)	0.09	−1.46	0.198
R^2^/R^2^ adjusted	0.471/0.451			
**Effect**	**IQCODE Score**			
	**Std. Beta**	**Standardized Std. Error**	**Statistic**	***p***
(Intercept)	0.00 (−0.22–0.22)	0.11	25.17	<0.001
IADL	−0.12 (−0.35–0.10)	0.11	−1.09	0.360
Geriatric Depression Scale	−0.11 (−0.35–0.13)	0.12	−0.92	0.360
FRAIL Scale	0.25 (0.01–0.49)	0.12	2.04	0.090
R^2^/R^2^ adjusted	0.077/0.041			

* *p* < 0.05 *** *p* < 0.001.

**Table A8:** Effect of demographic and selected baseline variables on 12-month mortality.

Effect	Deceased			
	Std. Beta	Standardized Std. Error	Statistic	*p*
(Intercept)	0.12 *** (0.04–0.30)	0.06	−0.53	0.595
Fasting Time Liquids	1.68 (0.94–3.06)	0.50	1.76	0.079
Postoperative Delirium	1.83 (0.40–8.06)	1.37	0.80	0.422
Sex (male)	0.53 (0.13–1.98)	0.36	−0.92	0.357
Age	1.06 (0.52–2.13)	0.37	0.16	0.870
CCI	1.80 (0.97–3.39)	0.56	1.89	0.059
Number of Medications	1.49 (0.72–3.29)	0.56	1.05	0.293
MoCA	0.55 (0.29–1.02)	0.18	−1.87	0.062
EQ-5D-5L	1.55 (0.82–2.94)	0.50	1.37	0.170
Neurodegenerative Disease (Yes)	0.87 (0.13–4.77)	0.78	−0.16	0.874
Depression (Yes)	3.18 (0.37–25.45)	3.37	1.09	0.275
Duration of Anesthesia	1.09 (0.50–2.15)	0.40	0.24	0.811
Hypotension	1.01 (0.51–1.80)	0.31	0.03	0.975
R^2^ Tjur	0.338			

*** *p* < 0.001.
